# Do Triathletes Periodize Their Diet and Do Their Mineral Content, Body Composition and Aerobic Capacity Change during Training and Competition Periods?

**DOI:** 10.3390/nu15010006

**Published:** 2022-12-20

**Authors:** Krzysztof Durkalec-Michalski, Natalia Główka, Paulina M. Nowaczyk, Anna Laszczak, Anna Gogojewicz, Joanna Suliburska

**Affiliations:** 1 Department of Sports Dietetics, Poznan University of Physical Education, 61-871 Poznań, Poland; 2 Department of Physiology and Biochemistry, Faculty of Physical Education and Sport, Charles University, 162 52 Prague, Czech Republic; 3 Department of Food and Nutrition, Poznan University of Physical Education, 61-871 Poznań, Poland; 4 Department of Human Nutrition and Dietetics, Poznań University of Life Sciences, 60-624 Poznań, Poland

**Keywords:** triathlon, nutrition assessment, aerobic capacity, elemental hair analysis

## Abstract

The triathlon is a demanding endurance multisport, which may strongly affect the nutritional status of athletes. The aim of this study was to find whether there are any differences in energy value and nutrient intake, body mass and body composition, aerobic performance and hair mineral status between training and competition periods and to assess whether there is a link between hair mineral content and physical capacity and nutrition. This observational study covered 20 triathletes aged 32 ± 7 years. The results of our study indicated performance improvement during the competition period (longer time to exhaustion (*p* = 0.025) and lower maximal oxygen uptake at the ventilatory threshold (%VO_2max_VT_; *p* = 0.047)). However, no differences were recorded in nutrition and body composition between two training vs. competition periods. There was a significant depletion in hair iron content during the competition period (*p* = 0.010). Furthermore, there were significant relationships between hair calcium content and absolute maximal oxygen uptake and %VO_2max_VT_ during the training period. It is necessary to introduce nutritional education in the group of triathletes focused on exercise-oriented nutritional periodization following the requirements of the training and competition periods, thus preventing the risk of nutrient deficiencies.

## 1. Introduction

The triathlon is a demanding endurance multisport, which is a combination of three consecutive disciplines, i.e., swimming, cycling and running, completed over a variety of distances. During a competition, there is only a brief transition period between these disciplines within a specific zone [1]. The overall duration of the event may last from 20 min up to even 8–9 h, depending on the many variations in distance, which demands the contribution and interaction of different energy systems and necessitates athletes having well-developed anaerobic qualities to meet specific requirements. In turn, during training, athletes focus on developing mainly aerobic capabilities, but also anaerobic power and capacity by high-intensity and speed work programmes [2].

The athletic success achieved in competitive sports is influenced by many factors, both exogenous and endogenous. Some of them, i.e., the training regime, nutrition, physical and physiological indicators, experience and technique, mental state and motivation, are shaped in the preparatory process by athletes [1,3,4,5]. Combining three different disciplines into one competition formula requires athletes to take up greater training volume and loads than training individual disciplines separately. Endurance training, which leads to changes in metabolism, respiratory, circulatory and muscular systems, is one of the most important parts of the training process for a triathlon. Adaptations of the organism to undertake long-term exercise allow for delaying the onset of fatigue while undertaking physical activity [1,3].

The ability to perform long-term efforts is determined to the greatest extent by the high aerobic capacity potential, the availability of energy substrates, enzyme activity, and the efficiency of thermoregulation and the cardiorespiratory system. This predictor is the basis for assessing an athlete’s fitness level and is shaped by genetic factors, training regime, nutrition and body composition, among other factors [1,6]. Furthermore, it should be emphasised that intensive training, nutritional discrepancy and mental stress, cause muscle fatigue and damage, as well as oxidative stress, which shape the specific nutritional needs of physically active people [1,6].

For competitive athletes undergoing strenuous training sessions and genetically predisposed to succeed in their sport, proper nutrition determines the ability to participate in high-impact sports training and may make the difference between victory or failure [7]. Proper diet and supplementation significantly support training-induced stimulation and exercise adaptation, as they support the process of generating energy during physical exertion and post-recovery efficiency (including, i.e., energy substrates’ resynthesis after the regeneration of damaged tissues) [7]. Although the aforementioned role of sports nutrition is widely known, the current state of knowledge about the nutrition of various groups of athletes shows the prevalence of qualitative nutritional mistakes and the limited scale of implementation of the recommendations of the Swiss nutrition pyramid. Results from the study conducted on a representative group of Polish athletes revealed the main mistakes concerning insufficient frequency of consuming different groups of food products, a high prevalence of irregular meals and non-limiting intake of energy drinks and sweetened beverages [8]. Furthermore, another important aspect from the point of view of sports training is to achieve and maintain an adequate nutritional status. In this respect, the basic factor providing information about the nutritional status is body composition, and the analysis of the concentration of selected clinically relevant biochemical markers (e.g., in blood, saliva, urine and hair) should be indicated at the forefront. Monitoring the body composition indicators of athletes allows, inter alia, to assess the effectiveness of dietary and training interventions. On the other hand, manipulation of the body composition allows for support of physical performance, adaptation to the requirements of a given sport discipline and taking on high-intensity exercise loads [9,10,11,12]. Similarly, an important element is also the nutritional status assessment involving the analysis of selected nutrients’ balance—especially over a longer perspective and time (which is possible, for example, by elemental hair analysis).

Finally, it should be pointed out that the above-mentioned elements are interrelated and may determine the proper body homeostasis and training process of an athlete, although their joint analysis is rarely carried out. Therefore, the objective of this study was to assess differences in nutrition and nutritional status related to body composition and hair mineral content, as well as physical capacity between two periods of macrocycles (training and competition), which differ between each other based on focusing on developing different qualities and capabilities in triathletes. To specify, body mass and body composition, nutritional value of a habitual diet, indices of aerobic capacity, as well as hair content of selected minerals were assed in both training and competition periods, to assess whether triathletes modify their diet in different athletic macrocycles and if this aspect may influence the changes in nutrition status and aerobic capacity and if changes in nutritional status may indicate possible variations in nutritional requirements during different macrocycle periods. Subsequently, the regression analysis between hair mineral content vs. energy value and nutrient intake and vs. indices of aerobic capacity was performed within both evaluated periods to verify the impact of nutrition on nutritional status and the link between nutritional status and aerobic capacity. It was hypothesized that there would be differences between two macrocycles and that a correlation between hair mineral content of athletes and nutrition, aerobic capacity and body composition will be vital.

## 2. Materials and Methods

### 2.1. Study Group

**Figure 1:**
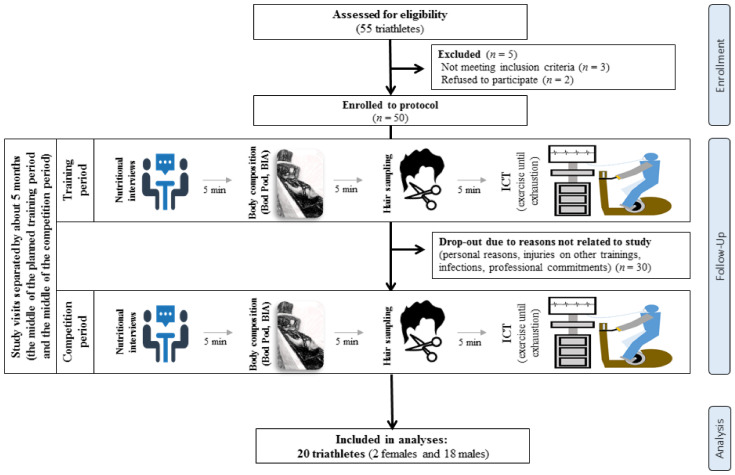
Flow chart of the study design.

Initially, the study group consisted of 50 triathletes (Figure 1). Due to injuries or other health problems disallowing the athlete to train or to take part in both periods of the study phases, refusal to continue participation in the research, or the suspicion of the authors of this paper of the athlete’s non-compliance with the recommendations that formed the basis for maintaining the appropriate conditions of the research conducted, the number of athletes participating in the research decreased. Finally, the study group consisted of 20 athletes (2 females and 18 males) practicing for triathlons at the moderate (but competitive) and professional levels. The average training experience of athletes was 8.5 ± 4 years, and the average age was 32 ± 7 years (Table 1). All athletes declared good health and voluntary willingness to participate in the research. The study protocol was reviewed and approved by the Bioethics Committee of the Poznan University of Medical Sciences, reference numbers 681/16 and 683/16 (10 November 2016). All study participants gave written informed consent. All procedures were carried out in accordance with the ethical standards of the Helsinki Declaration of 1975.

### 2.2. Study Design

This observational study was conducted during two triathlon-specific training macrocycles, i.e., the preparatory training period of February–March and the competition period of June–August.

### 2.3. Anthropometric and Body Composition Measurements

The study group was informed that at least 24 h before the body composition analysis, they should refrain from strenuous exercise and alcohol consumption and limit their coffee intake. The participants were also asked to pay special attention to proper hydration. They were aware that inadequate hydration due to excessive fluid loss or improper fluid intake can result in unreliable results from body composition analysis by bioelectrical impedance analysis (BIA). Males were also asked to shave their facial hair due to the effect of body hair on the results of body composition analysis by plethysmography.

Prior to body composition analysis, height and body mass were measured in duplicate each time the participants visited the laboratory using a calibrated scale with a stadiometer (WPT 60/150 OW, Radwag^®^, Radom, Poland) in a fasted state to the nearest 0.1 kg and 0.1 cm, respectively. The total body water and hydration level were assessed by bioelectric impedance with Bodystat 1500 (Bodystat Inc., Douglas, UK) and via urine specific gravity measurement with URYXXON^®^ Relax (Macherey-Nagel, Düren, Germany); values < 1.020 indicated proper hydration. Only properly hydrated participants were approved for testing. During the bioimpedance analyses, the recommended measurement conditions were strictly followed [13]. Fat-free mass and fat mass were assessed by air displacement plethysmography (Bod Pod^®^, Cosmed, Rome, Italy) as described previously [14,15]. The excellent repeatability and reliability of the applied parameters in the current study methods for body composition analysis were previously insightfully evaluated and are published elsewhere [15].

### 2.4. Nutritional Assessment

The assessment of the diet was made on the basis of the open-ended dietary recording method from the period of three consecutive days before each test round. Participants were trained in the dietary recording method by a dietitian. They were also provided with special food diary forms and a photo album of food products and dishes prepared by the National Food and Nutrition Institute in Warsaw [16]. At each testing visit, a face-to-face dietary interview with each participant was undertaken, to discuss any doubts regarding dietary recording diaries. The quantitative analysis of the composition of daily food rations was carried out using the Dietetyk 2011 software package (Jumar, Poland), which uses a database developed by the National Food and Nutrition Institute in Warsaw [17].

### 2.5. Aerobic Capacity Assessment

An incremental cycling test (ICT) was conducted to evaluate the aerobic fitness and capacity of the study group during two periods of the training macrocycles. Before ICT, athletes were advised to consume a standard pre-workout meal at 2 h before performing the exercise test. Prior to the test, athletes were asked to apply adequate recovery and to resign from any training the day before the test.

ICT was carried out on the Kettler X1 cycloergometer (Kettler, Ense-Parsit, Germany) using a Quark CPET ergospirometer (Cosmed, Rome, Italy) to assess aerobic fitness and capacity according to the procedure described previously [15]. Before the test, a mask was applied on the athlete’s face and a belt, with electrodes for heart rate (HR) measurement was placed on the chest. The athlete’s task was to maintain a constant cadence of ~70 ± 5 RPM during the exercise test. Participants started with a low load, i.e., 100 W for males and 75 W for females, which increased by 25 W every 1.5 min of exercise. The test continued until the subjective feeling of exhaustion of the athlete, i.e., refusal to undertake further physical exertion. During the test, selected respiratory and cardiovascular indices were analysed, i.e., minute oxygen uptake (VO_2_), minute ventilation (VE) and HR. Aerobic fitness and capacity was expressed by the maximal oxygen uptake (VO_2max_) and ventilatory threshold (VT) markers.

From the collected data, time to exhaustion (T_exh_), VO_2max_, and VO_2_ at VT (VO_2VT_), percentage of VO_2max_ at VT (%VO_2max_VT_), HR maximum (HR_max_) and at VT (HR_VT_), as well as workload expressed in watts at VT (W_VT_) were determined. The excellent repeatability and reliability of the aerobic capacity methodology were previously evaluated, and the results are published elsewhere [15].

### 2.6. Hair Sampling, Preparation and Analysis of the Mineral Content

Hair samples were collected during each test round. Proximal parts (1–2 cm in length, total weight of 0.5 g) of occipital scalp hair strands were collected after cutting hair using ethanol-precleaned stainless steel scissors. Hair samples were stored in a laboratory at room temperature until analysis.

The collected hair samples were washed with acetone and deionized water with subsequent drying on air to a stable weight. Hair samples were mineralized using a microwave digestion system (Speedwave Xpert, Berghof, Eningen, Germany) by digesting in 65% (*w*/*w*) spectra pure HNO_3_ (Merck, Kenilworth, NJ, USA).

The analysis of copper (Cu), iron (Fe), zinc (Zn), calcium (Ca) and magnesium (Mg) levels in the samples was performed using an AAS-3 spectrophotometer (Carl Zeiss, Germany). The accuracy of the assay was 94%, 94%, 102%, 95% and 91% for magnesium, iron, copper, zinc and calcium, respectively, as verified by certified reference materials (Human Hair NCS DC73347a, LGC). The results of hair analysis were expressed as μg/g for all elements.

### 2.7. Statistical Analysis

All variables were checked for normal distribution using the Shapiro–Wilk test. Results are presented as mean ± 1 standard deviation (SD) and 95% confidence interval (95% CI). Comparisons between two training periods were performed using *T*-test for dependent samples (normal distribution of the data) or Wilcoxon signed-rank test (non-normal distribution of the data). The relationships between nutrition or physical capacity indices and the content of minerals in collected hair samples were analysed using Spearman’s rank correlation (due to non-normal distribution of at least of one variable in all the analysis). Statistical significance was set at *p* < 0.05. Data were analysed using STATISTICA 13.3 (StatSoft Inc., Tulsa, OK, USA) software.

## 3. Results

### 3.1. Anthropometric and Body Composition Results

Body mass slightly decreased from 80.5 ± 14.4 kg in the training period to 78.6 ± 11.2 kg in the competition period, especially due to the insignificant reduction of body fat (Table 2). Furthermore, other body composition indices were not different between training and competition periods. However, it should be mentioned that the average % of fat mass was above the reference values for triathletes (5–12%) [18]. The average water content in the athletes’ bodies was within the generally accepted reference limits (45–75%) [19], both in the training and competition period, and amounted to 58.7 ± 5.1 and 59.9 ± 4.1%, respectively (Table 2).

### 3.2. Nutritional Assessment Results

In comparison to recommendations, the studied group provided lower energy (recommended ≥40 kcal/kg_BM_/day) and carbohydrate (recommended ≥6 g/kg_BM_/day) intake, as well as vitamin E (recommended 15 mg/day; 85% of participants not meeting the recommendations in both periods) and folic acid (recommended 400 µg/day; 85 and 80% of participants not meeting the recommendations in training and competition period, respectively) during both periods. Moreover, during the training period, athletes did not meet their requirements for vitamin C (recommended 75 mg/day; 60% of athletes not meeting the recommendations) and during the competition period for calcium (recommended 1000 mg/day; 65% of participants not meeting the recommendations) [7,20,21]. Nevertheless, statistical analysis showed no significant differences in energy and nutrient intake between the two periods of the training macrocycles (Table 3 and Table 4). The assessment of nutritional value of the diet indicates that studied triathletes did not periodize their nutrition depending on the training cycle (training vs. competition period).

### 3.3. Aerobic Fitness and Capacity

During the competition period, athletes performed the incremental exercise test significantly longer (*p* = 0.025) compared to the training period. In addition, %VO_2max_VT_ decreased (*p* = 0.047) during the competition period. However, the lack of changes in relative VO_2_ uptake at VT may limit clinical significance of the aforementioned %VO_2max_VT_ changes. Moreover, there were also no significant improvement in the rate of other cardiorespiratory maximal and threshold values (Table 5).

### 3.4. Hair Mineral Content Results

**Figure 2:**
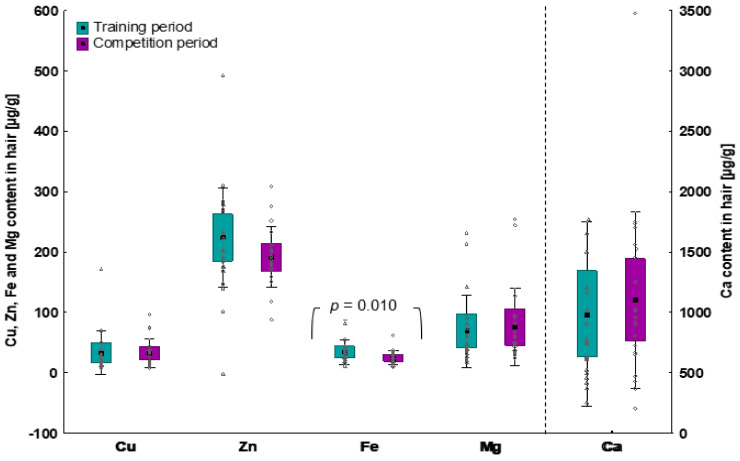
The content of chosen minerals in hair of triathletes during the training and competition period. Data are expressed as means (square), 95% CI (box), 95% CI + SD (whisker), raw data. Data analysed by Wilcoxon signed-rank test.

The average iron content was significantly lower at the competition period (~28.2% lower) compared to the training period. There was no significant difference in the content of the remaining analysed minerals between studied macrocycle periods (Figure 2).

### 3.5. Correlations between Analysed Markers

#### 3.5.1. Correlation between Hair Mineral Content vs. Energy and Selected Nutrients Intake

The associations between hair mineral content and nutritional value of habitual diet occurred solely during the training period. There were negative relationships between hair zinc content and intake of protein (as expressed as g/day *<r* = −0.514, *p* = 0.020> and g/kg_BM_ <*r* = −0.535, *p* = 0.015>; Table 6), polyunsaturated fatty acids (*r* = −0.492, *p* = 0.028), vitamin E (*r* = −0.570, *p* = 0.009; Supplementary Table S1) and vitamin B_3_ (*r* = −0.549, *p* = 0.012; Supplementary Table S1). There was positive association between hair copper content and intake of fat (as expressed as g//kg_BM_ < *r* = 0.534, *p* = 0.015; Table 6). There were no significant relationships between hair mineral content and minerals intake at any of the macrocycle periods (Table 7).

#### 3.5.2. Correlation between Hair Mineral Content vs. Aerobic Fitness and Capacity

During the training period, there was a significant positive relationship between hair calcium content and absolute VO_2max_ (*r* = 0.481, *p* = 0.032) and a negative relationship between hair calcium content and %VO_2max_VT_ (*r* = −0.571, *p* = 0.008; Table 8). However, during the competition period, there were no associations between hair mineral content and indices of aerobic capacity.

## 4. Discussion

The results of the current study indicated significantly longer T_exh_ and lower %VO_2max_VT_, as well as lower hair iron content during the competition period compared to the training period, but no significant differences in nutrition, body composition and remaining hair minerals content between two periods of macrocycles in the studied group of triathletes. During the training period there were significant correlations only between the content of: (1) zinc vs. protein, polyunsaturated fatty acids, vitamins E and B_3_; (2) copper vs. fat intake; and (3) calcium vs. absolute VO_2max_ and %VO_2max_VT_. During the competition period there were no significant correlations between hair minerals content and any of the indices of habitual diet or aerobic capacity.

In the approach of holistic and interdisciplinary sports training, body composition especially reflects the nutritional status of athletes. Thus, body composition monitoring makes it possible to assess the effectiveness of the undertaken nutritional and training interventions. Deliberate manipulation of body composition at different periods of the annual training macrocycles allows, among other things, to support physical performance, meet the physical demands of a given sport and meet significant exercise loads. Elite endurance athletes are typically lean and have relatively low body mass as a result of the genetic factors that have predisposed them to being successful in their sport, as well as the modifying effects of training and diet [1]. The total body fat percentage is between 5% and 10% for young athletic males and between 8% and 15% for young athletic females, whereas it is 5–12% and 10–15% for triathletes, respectively [18]. The average body fat percentage for high-level triathletes is 11%. Excess fat mass seems to reduce performance in triathlons, above all in the cycling and running segments [22]. During the general preparation phase of each season, optimal energy availability should be prioritised, with the athletes being ∼2–4% over their ideal “race weight” and body fat levels [1]. Nevertheless, our results showed higher values of percentage of body fat and no significant changes between macrocycles in studied triathletes.

There is a clear interaction between body composition and physical capacity of triathletes [1]. A study involving 184 triathletes showed a correlation between body mass and body fat percentage and athletic performance and interval effort times obtained by athletes. Lower body mass and body fat percentage are associated with faster running times, as well as overall times obtained at competitions [23,24,25,26]. It is known that adipose tissue does affect the power generated by the athlete, so an increase in its proportion in the athlete’s body will be accompanied by an obvious decrease in the value of the relative power output, e.g., at maximal intensity or VT. Changes in body composition will therefore affect not only oxygen uptake, but also the athlete’s power and/or strength. A study involving 15 elite triathletes [27] showed that the maximum power that athletes are able to achieve during exercise also has an impact on the performance of triathletes. Moreover, the same authors [27] concluded that the economy of energy substrates oxidation and the movements performed by athletes in the cycling stage are also crucial for long distances. However, the position of the American Society of Sports Medicine indicates that, for disciplines in which the athlete must traverse longer distances, a more important aspect than the athlete’s maximal power output will be the optimization of power output, especially related to body mass [28,29]. In our study, significant differences between macrocycles relating to physical capacity applied to T_exh_ and %VO_2max_VT_. Therefore, periodization in terms of most of the values of aerobic capacity indices did not occur—similarly as it was revealed in the field of nutrition.

Properly designed diet is one of the foundations of good training. Sufficiently meeting energy and nutrients requirements may aid and augment physiological training adaptations. Avoiding energy deficiency during training may prevent loss of muscle mass, bone mineral density, strength, decrease susceptibility to illness and injuries, disturbances in immune, endocrine and reproductive functions and prevalence of overtraining [7]. In spite of recognition by other researchers of the influence of nutrition on training adaptations, our research did not reveal any nutrition periodization practices in the training and competition periods in the studied group of triathletes. This seems disturbing considering that athletes in both periods followed their usual diet, which they also considered an effective part of their training process. It has to be underlined that educating athletes and coaches about nutrition and how to periodize diet to optimize training, exercise performance and recovery are key areas of involvement for sport dietitians and nutritionists. Taking into account the unique energy and biomechanical requirements, the triathlon is a discipline in which the implementation of strategic nutritional interventions at different times of the training and competition macrocycles seems to determine training, support adaptation, recovery and physical capacity [30].

However, for enhancement of athletic exercise performance and optimization of training adaptations, it is important to not only meet energy and macronutrients requirements but take care of overall nutrition (such as the intake of vitamins and minerals) [30].

The importance of minerals in terms of influencing sports capabilities seems to be well documented [31,32,33,34,35]. However, there is the lack of holistic approaches and assessments of the degree of nutrients’ (especially minerals) intake in the customary diets and mineral status evaluation combined with physical capacity monitoring in different athletic macrocycles.

Although in our study there were no differences in dietary intake of minerals and vitamins in the study group between the training and competition period, there were several deficiencies. In both training periods, energy value of the diet of studied triathletes, as well as intake of carbohydrates and folic acid, were below the recommendations. Moreover, the intake of vitamin C in the training period and calcium in the competition period was insufficient. A study by Muros et al. on triathletes [36] showed similar results concerning an inadequate amount of carbohydrates, which also is in line with other reports [37]. In addition, the discussed study [36] showed deficiencies in vitamin D and E.

It should be mentioned that hair mineral analysis may be one of the additional methods of assessing nutritional status and allows monitoring the elemental balance of the body [38]. Hair mineral analysis has also become an interesting diagnostic tool in biomonitoring of exposure to toxic elements and in the assessment of health. It is important to underline that there are only sparse studies on hair mineral content in athletes, but many of them assessed the correlations carried out in the current study. A study by Zaisteva and Zaitsev [39] showed an increase in the content of macronutrients Ca, Mg, P, K and Na in young wrestlers’ bodies. A study by Zaborova et al. [40] showed that wrestlers, in comparison with the control group, had higher levels of many trace elements in hair, such as lithium, beryllium, boron, sodium, aluminium, calcium, potassium, vanadium, magnesium, cobalt, copper, germanium, rubidium, strontium, cadmium, antimony, caesium, barium and thallium. In turn, Zaitseva et al. [41] showed that students with increased physical activity had decreased hair copper, vanadium, bismuth and mercury content in comparison to students with low physical activity levels. Nabatov et al. [42] showed that female athletes had increased levels of selenium, and field hockey players had higher levels of zinc in hair. Nevertheless, there are no studies correlating minerals content in hair and physical capacity. It is difficult to compare results from hair mineral analysis to serum or urine content of elements, because there is no clear consensus on the relationship between these contents.

In the current study, hair iron content was significantly lower in the competition period compared to the training period. Simultaneously, habitual iron intake remained stable within the studied periods of training and competition macrocycles. Thus, this serves as a clear and evident indication for triathletes to periodize their nutrition, at least with respect to iron intake. Due to increased turnover in response to high training loads, higher intake of iron must be recommended right before and during the competition period.

It is important to note that the current study revealed a few significant correlations between the content of minerals in hair vs. nutrition and aerobic capacity indices. Interestingly, in the training period, there was: (1) a significant positive relationship between hair calcium content and absolute VO_2max_; and (2) a significant negative relationship between hair calcium content and %VO_2max_VT_. This suggests that the adequate nutritional state regarding Ca storages may play a remarkable role in determining the level of physical capacity. Although a few indices of aerobic capacity (i.e., an increase in absolute VO_2max_ and a decrease in %VO_2max_VT_) pointed at improved performance during the competition period, this improvement was not supposed to be due to improvement in diet nutritional content (while no differences in the indices of diet quality were observed), but probably due to training regime only. Taking into account: (1) the relationships between hair Ca content and indices of aerobic capacity during the training period; (2) the lack of improvement in the nutritional value of diet during the competition period; and (3) the improvement in some aerobic capacity indices during the competition period—it should be expected that periodization in food consumption, reflected by higher intake of some crucial nutrients (e.g., calcium and iron) during the competition period, would result in even greater than currently observed improvements in aerobic capacity. The above mentioned data clearly pointed at an urgent need for a holistic and interdisciplinary training approach.

Furthermore, the average dietary iron intake was lower than the recommended level for some of the studied triathletes in both macrocycles. The deterioration of the iron nutritional status could have resulted from a reduced and inadequate energy intake in the training period. The lower supply, including lower energy availability, could therefore indicate that triathletes in the competition period are more prone to iron deficiency. For this reason, a higher supply of Fe, especially in a negative energy balance, could be highly recommended. In addition, it should be remembered that an important aspect of the bioavailability of minerals are their interactions with each other. In this regard, vitamin C may influence iron metabolism.

Finally, in the interpretation of the results, certain limitations of the presented studies should be taken into account. These include the relatively limited sample size, the lack of discipline-specific performance analysis and only two measurement time-points (one during the training period and one during the competition period). It needs to be mentioned that the number of female participants in the studied group was small (*n* = 2). Thus, it was impossible either to perform the analysis separately for genders or to introduce gender as a confounding factor into analyses (also taking into account the types of the statistical tests applied). Still, this is a clear indication for an urgent need to extend this type of evaluation on female populations of triathletes. It is particularly important, taking into account an increased requirement for certain nutrients, i.e., iron, in female athletes. The strengths, however, include exemplary research methods, homogeneity of the athletes and the fact that the same participants were tested in two different periods (training and competition). Furthermore, it is also worth highlighting that the methodology and results obtained in the current study distinctly indicate its novelty in the field of sports nutrition, especially the part that focused on athletes’ mineral status during different times of the annual macrocycles. To sum up, assessing differences in several aspects, such as nutrition, nutritional status, body composition, physical capacity, hair mineral content and relationships between the aforementioned, during two periods of macrocycles in triathletes, may determine future possibilities that can be translated into real-world cooperation between specialists and high-level professional athletes.

## 5. Conclusions

Although there were no differences in energy and nutritional values of habitual diets in studied triathletes between training and competition periods, a substantial depletion in hair iron content occurred in the competition period. This may indicate increased nutritional needs during the competition period. There was an improvement in selected indices of aerobic capacity during the competition period. There were significant relationships between hair calcium content and some indices of aerobic capacity during the training period (positive with absolute maximal oxygen uptake and negative with percent of maximal oxygen uptake at ventilatory threshold). The results of the current study revealed lack of dietary periodization according to training macrocycles. Moreover, the recognized nutritional deficiencies pointed at the urgent necessity of introducing nutritional education focused on periodization of energy and macro- and microelements intake in the group of triathletes. Finally, this study should be treated as preliminary, which, due to the above-mentioned aspects, emphasizes the need for conducting further investigations in this area, especially in female triathletes.

## Figures and Tables

**Table 1:** Characteristics of the tested group after enrolment in the studies.

Variable		Mean ± SD	Min–Max
Age	[years]	32 ± 7	20–40
Body mass	[kg]	80.5 ± 14.4	55.8–123.2
Body height	[cm]	179 ± 9	157–190
Training experience	[years]	8.5 ± 4.0	2–17
Weekly training length	[h]	11.0 ± 5.5	1–20

**Table 2:** Assessment of the anthropometric and body composition indices of athletes during the training and competition period.

		Training Period		Competition Period		
Variable		Mean ± SD	95% CI	Mean ± SD	95% CI	*p*
Body mass	[kg]	80.5 ± 14.4	73.7–87.2	78.6 ± 11.2	73.3–83.8	0.078 ^†^
Fat mass	[kg]	15.1 ± 9.0	10.9–19.4	12.9 ± 5.4	10.2–15.6	0.157 ^§^
	[%]	18.1 ± 8.2	14.3–22.0	16.4 ± 6.3	13.3–19.6	0.244 ^†^
Fat free mass	[kg]	65.6 ± 9.5	61.2–70.1	65.5 ± 10.3	60.4–70.6	0.557 ^§^
	[%]	81.9 ± 8.2	78.0–85.7	83.6 ± 6.3	80.4–86.7	0.244 ^†^
Total body water	[L]	46.9 ± 6.8	43.7–50.1	47.1 ± 7.2	43.7–50.4	0.940 ^§^
	[%]	58.7 ± 5.1	56.4–61.1	59.9 ± 4.1	58.0–61.9	0.125 ^†^

^†^ data analysed by *T*-test for dependent samples; ^§^ data analysed by Wilcoxon signed-rank test.

**Table 3:** Daily energy and macronutrients intake in the diet of triathletes during the training and competition period.

		Training Period		Competition Period		
Variable		Mean ± SD	95% CI	Mean ± SD	95% CI	*p*
Energy intake	[kJ/day]	10,930 ± 3271	9400–12,461	10,533 ± 3384	8949–12,117	0.455 ^§^
	[kcal/day]	2601 ± 794	2229–2972	2535 ± 812	2155–2915	0.455 ^§^
	[kcal/kg_BM_/day]	33 ± 11 *	28–38	33 ± 11 *	28–38	0.940 ^§^
Carbohydrate	[g/day]	343 ± 127	283–402	345 ± 119	290–401	0.823 ^§^
	[g/kg_BM_/day]	4.3 ± 1.7 *	3.5–5.1	4.4 ± 1.5 *	3.7–5.1	0.455 ^§^
	[% energy]	51. 8 ± 9.0	47.6–56.0	54.8 ± 9.1	50.5–59.0	0.351 ^§^
Dietary fibre	[g/day]	29.2 ± 12.5	23.4–35.0	29.5 ± 15.3	22.3–36.6	0.940 ^§^
Protein	[g/day]	116 ± 39	98–135	114 ± 44	94–135	0.411 ^§^
	[g/kg_BM_/day]	1.5 ± 0.5	1.2–1.7	1.5 ± 0.5	1.2–1.7	0.575 ^§^
	[% energy]	18.2 ± 4.4	16.2–20.3	18.4 ± 4.9	16.1–20.6	0.370 ^§^
Fat	[g/day]	94 ± 33	79–110	92 ± 46	70–113	0.601 ^§^
	[g/kg_BM_/day]	1.2 ± 0.5	1.0–1.4	1.2 ± 0.6	0.9–1.5	0.765 ^§^
	[% energy]	33.2 ± 7.5	29.7–36.7	31.8 ± 8.7	27.8–35.9	0.852 ^§^
SFA	[g/day]	30.1 ± 13.2	23.9–36.2	30.9 ± 18.1	22.4–39.4	0.970 ^§^
MUFA	[g/day]	34.8 ± 14.6	27.9–41.6	35.2 ± 21.2	25.3–45.1	0.823 ^§^
PUFA	[g/day]	18.1 ± 11.1	12.9–23.3	14.6 ± 14.2	8.0–21.3	**0.028** ^§^
Cholesterol	[mg/day]	357 ± 138	293–422	417 ± 206	321–514	0.259 ^†^

SFA, saturated fatty acids; MUFA, monounsaturated fatty acids; PUFA, polyunsaturated fatty acids. * below the recommendations [7,20,21]; ^†^ data analysed by *T*-test for dependent samples; ^§^ data analysed by Wilcoxon signed-rank test.

**Table 4:** Vitamins and minerals intake in the diet of triathletes during the training and competition period.

		Training Period		Competition Period			
Variable		Mean ± SD	95% CI	Mean ± SD	95% CI	*p* ^§^	Recommended Dietary Allowance (Males/Females)
Vitamin A	[µg/day]	1115 ± 675	799–1431	1704 ± 1662	926–2482	0.108	900/700
Vitamin D	[µg/day]	5.3 ± 4.0	3.4–7.2	5.5 ± 3.8	3.7–7.3	0.823	5
Vitamin E	[mg/day]	11.7 ± 6.8 *	8.5–14.9	13.0 ± 12.5 *	7.2–18.9	0.881	15
Vitamin C	[mg/day]	73.9 ± 52.7 *	49.2–98.5	110.3 ± 144.5	42.7–178.0	0.135	90/75
Vitamin B_1_	[mg/day]	1.7 ± 0.7	1.4–2.0	2.5 ± 3.9	0.7–4.3	0.391	1.2/1.1
Vitamin B_2_	[mg/day]	2.2 ± 0.8	1.8–2.6	3.0 ± 4.0	1.2–4.9	0.737	1.3/1.7
Vitamin B_3_	[mg/day]	21.8 ± 9.7	17.3–26.3	21.5 ± 10.8	16.4–26.5	0.627	16/14
Vitamin B_6_	[mg/day]	2.8 ± 1.0	2.3–3.2	3.6 ± 4.7	1.4–5.8	0.575	1.3
Vitamin B_12_	[µg/day]	5.5 ± 3.3	4.0–7.1	7.1 ± 5.6	4.5–9.8	0.970	2.4
Folic acid	[µg/day]	295 ± 112 *	242–348	349 ± 236 *	238–459	0.526	400
Sodium (Na)	[mg/day]	2460 ± 1393	1809–3112	2330 ± 1343	1702–2958	0.654	500
Potassium (K)	[mg/day]	4130 ± 1571	3394–4865	4019 ± 1480	3327–4712	0.970	2000 ^a^
Calcium (Ca)	[mg/day]	1064 ± 661	754–1373	993 ± 569 *	727–1260	0.681	1000
Phosphorus (P)	[mg/day]	1966 ± 679	1648–2283	1884 ± 672	1570–2199	0.455	700
Magnesium (Mg)	[mg/day]	470 ± 205	374–566	486 ± 281	355–618	0.627	420/320
Iron (Fe)	[mg/day]	15.7 ± 6.3	12.7–18.6	17.1 ± 8.5	13.1–21.1	0.455	8/18
Zinc (Zn)	[mg/day]	14.6 ± 5.5	12.1–17.2	15.9 ± 8.9	11.7–20.0	0.654	11/8
Copper (Cu)	[mg/day]	1.8 ± 1.0	1.4–2.3	2.0 ± 1.3	1.4–2.6	0.575	0.9
Manganese (Mn)	[µg/day]	7.3 ± 4.8	5.1–9.6	7.4 ± 5.1	5.1–9.8	0.709	2.3/1.8 ^b^

^§^ data analysed by Wilcoxon signed-rank test; * below the requirements [7,21]; ^a^ estimated minimum requirement [7,21]; ^b^ recommended consumption level used in standards for the Polish population [7,21].

**Table 5:** Aerobic capacity during the training and competition period.

			Training Period		Competition Period		
	Variable		Mean ± SD	95% CI	Mean ± SD	95% CI	*p*
Maximal values	Time to exhaustion	[s]	940 ± 156	868–1013	999 ± 190	910–1088	**0.025 ^†^**
	Absolute maximal oxygen uptake	[mL/min]	4204 ± 633	3908–4501	4253 ± 781	3888–4619	0.601 ^§^
	Relative maximal oxygen uptake	[mL/kg_BM_/min]	52.9 ± 7.7	49.3–56.5	54.1 ± 7.5	50.6–57.7	0.346 ^†^
	Maximum heart rate	[bpm]	181 ± 10	176–185	181 ± 9	177–185	0.744 ^†^
Threshold values	Time to ventilatory threshold	[s]	696 ± 146	628–765	725 ± 149	655–794	0.243 ^†^
	Absolute power output at ventilatory threshold	[W]	283 ± 47	261–304	288 ± 50	264–311	0.456 ^§^
	Relative power output at ventilatory threshold	[W/kg_BM_]	3.5 ± 0.5	3.3–3.8	3.7 ± 0.6	3.4–4.0	0.126 ^†^
	Absolute oxygen uptake at ventilatory threshold	[mL/min]	3660 ± 459	3445–3874	3543 ± 641	3243–3844	0.210 ^†^
	Relative oxygen uptake at ventilatory threshold	[mL/kg_BM_/min]	46.4 ± 6.7	43.3–49.5	45.3 ± 7.1	42.0–48.7	0.366 ^†^
	Heart rate at ventilatory threshold	[bpm]	165 ± 11	160–170	164 ± 10	159–169	0.645 ^†^
	Percent of maximal oxygen uptake at ventilatory threshold	[%]	87.9 ± 5.9	85.2–90.7	83.8 ± 6.9	80.6–87.1	**0.047 ^†^**

^†^ data analysed by *T*-test for dependent samples; ^§^ data analysed by Wilcoxon signed-rank test.

**Table 6:** Spearman correlation coefficient between hair mineral content and intake of macronutrients in the usual diet of triathletes.

			Training Period					Competition Period				
Diet/Hair			Cu [µg/g]	Zn [µg/g]	Fe [µg/g]	Ca [µg/g]	Mg [µg/g]	Cu [µg/g]	Zn [µg/g]	Fe [µg/g]	Ca [µg/g]	Mg [µg/g]
Energy	[kJ]	*r*	0.435	−0.232	0.120	0.093	0.220	0.251	0.242	−0.386	0.084	0.191
		*p*	0.056	0.326	0.613	0.696	0.352	0.286	0.304	0.092	0.724	0.420
	[kcal]	*r*	0.299	−0.238	0.044	0.123	0.203	0.284	0.272	−0.412	0.053	0.232
		*p*	0.200	0.313	0.855	0.605	0.391	0.225	0.246	0.071	0.826	0.326
	[kcal/kg_BM_]	*r*	0.329	−0.344	0.023	−0.044	0.116	0.078	0.056	−0.143	−0.123	−0.095
		*p*	0.156	0.127	0.925	0.855	0.627	0.743	0.816	0.548	0.605	0.691
Carbohydrate	[g]	*r*	0.173	−0.236	0.056	0.032	0.185	0.182	0.180	−0.391	−0.317	0.042
		*p*	0.466	0.316	0.816	0.895	0.435	0.443	0.446	0.088	0.173	0.860
	[g/kg_BM_]	*r*	0.245	−0.242	0.000	−0.086	0.011	0.056	0.005	−0.272	−0.376	−0.191
		*p*	0.298	0.304	1.000	0.719	0.965	0.816	0.985	0.246	0.102	0.420
	[% energy]	*r*	−0.188	−0.035	0.057	−0.182	0.041	−0.155	0.104	−0.238	−0.353	−0.026
		*p*	0.427	0.885	0.811	0.443	0.865	0.514	0.663	0.313	0.126	0.915
Lactose	[g]	*r*	−0.069	0.293	0.251	0.215	0.301	0.051	−0.212	−0.017	−0.024	0.289
		*p*	0.772	0.210	0.286	0.363	0.198	0.830	0.369	0.945	0.920	0.217
Dietary Fibre	[g]	*r*	0.008	−0.241	0.042	−0.092	0.189	0.047	0.123	−0.269	−0.355	−0.039
		*p*	0.975	0.307	0.860	0.701	0.424	0.845	0.605	0.251	0.125	0.870
Protein	[g]	*r*	0.174	**−0.514**	0.050	0.129	0.397	0.248	0.182	−0.346	−0.104	0.123
		*p*	0.462	**0.020**	0.835	0.587	0.083	0.292	0.443	0.135	0.663	0.605
	[g/kg_BM_]	*r*	0.368	**−0.535**	0.026	0.044	0.260	0.183	−0.110	−0.182	−0.189	−0.116
		*p*	0.110	**0.015**	0.915	0.855	0.268	0.439	0.645	0.443	0.424	0.627
	[% energy]	*r*	−0.215	−0.292	0.006	0.066	0.289	0.032	−0.147	0.174	0.003	−0.008
		*p*	0.363	0.212	0.980	0.782	0.217	0.895	0.535	0.462	0.990	0.975
Fat	[g]	*r*	0.436	−0.281	0.116	0.268	0.352	0.205	0.009	−0.089	0.188	0.036
		*p*	0.055	0.230	0.627	0.254	0.128	0.387	0.970	0.710	0.427	0.880
	[g/kg_BM_]	*r*	**0.534**	−0.379	0.108	0.137	0.244	0.208	−0.035	−0.033	0.171	−0.053
		*p*	**0.015**	0.099	0.650	0.565	0.301	0.380	0.885	0.890	0.470	0.826
	[% energy]	*r*	0.245	0.009	−0.128	0.152	−0.057	0.214	−0.018	0.062	0.319	0.042
		*p*	0.298	0.970	0.591	0.523	0.811	0.366	0.940	0.796	0.171	0.860
SFA	[g]	*r*	0.104	0.198	0.147	0.358	0.171	0.239	−0.008	−0.044	0.337	0.322
		*p*	0.663	0.402	0.535	0.121	0.470	0.310	0.975	0.855	0.146	0.166
MUFA	[g]	*r*	0.350	−0.101	−0.062	0.211	0.153	0.224	0.003	−0.099	0.221	0.044
		*p*	0.130	0.673	0.796	0.373	0.519	0.342	0.990	0.677	0.349	0.855
PUFA	[g]	*r*	0.281	**−0.492**	0.147	−0.150	0.192	0.146	0.296	−0.114	0.063	−0.012
		*p*	0.230	**0.028**	0.535	0.527	0.416	0.539	0.205	0.631	−0.012	0.960

SFA, saturated fatty acids; MUFA, monounsaturated fatty acids; PUFA, polyunsaturated fatty acids.

**Table 7:** Spearman correlation coefficient between hair mineral content and the intake of minerals in the usual diet of triathletes.

			Training Period					Competition Period				
Diet/Hair			Cu [µg/g]	Zn [µg/g]	Fe [µg/g]	Ca [µg/g]	Mg [µg/g]	Cu [µg/g]	Zn [µg/g]	Fe [µg/g]	Ca [µg/g]	Mg [µg/g]
Na	[mg]	*r*	0.020	−0.005	−0.014	0.251	0.260	−0.020	0.244	−0.230	−0.017	0.171
		*p*	0.935	0.985	0.955	0.286	0.268	0.935	0.301	0.329	0.945	0.470
K	[mg]	*r*	0.200	−0.269	0.057	0.068	0.269	0.176	0.341	−0.269	−0.134	−0.014
		*p*	0.398	0.251	0.811	0.777	0.251	0.458	0.141	0.251	0.574	0.955
Ca	[mg]	*r*	0.051	0.015	0.107	0.006	0.095	0.197	−0.322	−0.241	−0.041	0.108
		*p*	0.830	0.950	0.654	0.980	0.691	0.405	0.166	0.307	0.865	0.650
P	[mg]	*r*	0.150	−0.265	0.075	−0.017	0.259	0.242	0.230	−0.364	−0.209	0.111
		*p*	0.527	0.259	0.753	0.945	0.271	0.304	0.329	0.115	0.376	0.640
Mg	[mg]	*r*	0.113	−0.195	0.191	0.011	0.307	0.202	0.280	−0.352	−0.260	−0.023
		*p*	0.636	0.409	0.420	0.965	0.188	0.394	0.232	0.128	0.268	0.925
Fe	[mg]	*r*	0.024	−0.262	−0.120	−0.012	0.194	0.206	0.089	−0.111	−0.045	0.090
		*p*	0.920	0.265	0.613	0.960	0.413	0.384	0.710	0.640	0.850	0.705
Zn	[mg]	*r*	0.069	−0.158	−0.009	0.075	0.296	0.388	0.192	−0.340	−0.147	0.238
		*p*	0.772	0.506	0.970	0.753	0.205	0.091	0.416	0.143	0.535	0.313
Cu	[mg]	*r*	0.047	−0.280	0.110	−0.057	0.299	0.182	0.177	−0.195	−0.340	−0.053
		*p*	0.845	0.232	0.645	0.811	0.200	0.443	0.454	0.409	0.143	0.826
Mn	[µg]	*r*	−0.005	−0.117	0.280	−0.155	0.104	0.141	0.180	−0.253	−0.408	−0.092
		*p*	0.985	0.622	0.232	0.514	0.663	0.552	0.446	0.283	0.075	0.701

**Table 8:** Spearman correlation coefficient between hair mineral content and physical capacity of triathletes.

				Training Period					Competition Period				
	Variable			Cu [µg/g]	Zn [µg/g]	Fe [µg/g]	Ca [µg/g]	Mg [µg/g]	Cu [µg/g]	Zn [µg/g]	Fe [µg/g]	Ca [µg/g]	Mg [µg/g]
Maximal values	Time to exhaustion	[s]	*r*	0.056	−0.167	−0.101	0.167	0.093	0.361	0.344	−0.537	0.002	0.278
			*p*	0.816	0.482	0.673	0.482	0.696	0.118	0.137	0.015	0.995	0.235
	Absolute maximal oxygen uptake	[mL/min]	*r*	0.161	0.165	0.038	**0.481**	0.186	0.442	0.080	−0.444	0.167	0.272
			*p*	0.498	0.486	0.875	**0.032**	0.431	0.051	0.738	0.050	0.482	0.246
	Relative maximal oxygen uptake	[mL/kg_BM_/min]	*r*	0.328	−0.141	0.042	0.128	0.087	0.332	−0.101	−0.281	−0.268	−0.296
			*p*	0.158	0.552	0.860	0.591	0.715	0.152	0.673	0.230	0.254	0.205
	Maximum heart rate	[bpm]	*r*	0.288	0.050	0.269	0.225	0.012	0.180	−0.255	−0.142	0.087	−0.152
			*p*	0.219	0.833	0.252	0.340	0.960	0.448	0.279	0.549	0.714	0.522
Threshold values	Time to ventilatory threshold	[s]	*r*	−0.020	−0.193	−0.169	0.102	0.038	0.374	0.020	−0.383	−0.002	0.176
			*p*	0.935	0.416	0.476	0.668	0.872	0.105	0.935	0.095	0.992	0.459
	Absolute power output at ventilatory threshold	[W]	*r*	0.007	−0.088	−0.093	0.140	0.069	0.324	0.047	−0.331	−0.018	0.150
			*p*	0.977	0.712	0.697	0.557	0.774	0.163	0.845	0.154	0.940	0.527
	Relative power output at ventilatory threshold	[W/kg_BM_]	*r*	0.235	−0.198	−0.134	−0.065	−0.083	0.126	−0.086	−0.141	−0.266	−0.286
			*p*	0.319	0.402	0.574	0.787	0.729	0.596	0.719	0.552	0.257	0.222
	Absolute oxygen uptake at ventilatory threshold	[mL/min]	*r*	0.027	0.020	−0.080	0.280	0.111	0.311	0.164	−0.301	0.161	0.188
			*p*	0.910	0.935	0.738	0.232	0.640	0.182	0.490	0.198	0.498	0.427
	Relative oxygen uptake at ventilatory threshold	[mL/kg_BM_/min]	*r*	0.099	−0.209	−0.131	−0.214	−0.161	0.114	−0.042	−0.138	−0.189	−0.296
			*p*	0.677	0.376	0.582	0.366	0.498	0.631	0.860	0.561	0.424	0.205
	Heart rate at ventilatory threshold	[bpm]	*r*	0.136	−0.069	0.188	0.125	0.054	0.254	−0.296	−0.098	0.115	−0.043
			*p*	0.567	0.772	0.428	0.599	0.820	0.280	0.205	0.681	0.631	0.857
	Percent of maximal oxygen uptake at ventilatory threshold	[%]	*r*	−0.424	−0.203	−0.105	**−0.571**	−0.217	−0.319	0.002	0.371	0.248	0.060
			*p*	0.062	0.391	0.659	**0.008**	0.359	0.171	0.995	0.107	0.292	0.801

## Data Availability

The datasets used and/or analysed during the current study are available from the corresponding author on reasonable request.

## Supplementary Materials

The following supporting information can be downloaded at: https://www.mdpi.com/article/10.3390/nu15010006/s1, Table S1: Spearman correlation coefficient between hair mineral content and the intake of vitamins in the usual diet of triathletes
