# Supplementary material for: Do Triathletes Periodize Their Diet and Do Their Mineral Content, Body Composition and Aerobic Capacity Change during Training and Competition Periods?
Source: Nutrients. 2022 Dec 20;15(1):6. doi: 10.3390/nu15010006 (PMC9824709; doi:10.3390/nu15010006)
Supplement: Supplementary file 1 [file nutrients-15-00006-s001.zip › nutrients-2103252-supplementary.pdf]

Supplementary Table S1. Spearman correlation coefficient between hair mineral content and the intake of vitamins in the usual diet of triathletes

Diet/Hair			Training period					Competition period				
			Cu [μg/g]	Zn [μg/g]	Fe [μg/g]	Ca [μg/g]	Mg [μg/g]	Cu [μg/g]	Zn [μg/g]	Fe [μg/g]	Ca [μg/g]	Mg [μg/g]
**Vit A**	[μg]	*r*	-0.147	-0.146	-0.226	-0.006	-0.065	0.132	0.141	0.029	0.232	0.206
		p	0.535	0.539	0.339	0.980	0.787	0.578	0.552	0.905	0.326	0.384
**Vit D**	[mg]	*r*	0.065	-0.220	0.171	0.183	0.420	0.110	0.024	-0.017	0.089	-0.211
		p	0.787	0.352	0.470	0.439	0.066	0.645	0.920	0.945	0.710	0.373
**Vit E**	[mg]	*r*	0.311	**-0.570**	0.012	-0.021	0.238	-0.104	0.274	-0.021	-0.071	-0.171
		p	0.182	**0.009**	0.960	0.930	0.313	0.663	0.243	0.930	0.767	0.470
**Vit C**	[mg]	*r*	0.023	-0.072	-0.033	0.008	0.125	0.152	0.308	-0.257	0.101	-0.117
		p	0.925	0.762	0.890	0.975	0.600	0.523	0.186	0.274	0.673	0.622
**Vit B_1**	[mg]	*r*	-0.054	-0.214	-0.202	-0.012	0.168	0.326	0.180	-0.427	-0.029	0.183
		p	0.821	0.366	0.394	0.960	0.478	0.160	0.446	0.060	0.905	0.439
**Vit B_2**	[mg]	*r*	0.053	-0.110	0.029	0.170	0.409	0.395	-0.137	-0.062	-0.059	0.171
		p	0.826	0.645	0.905	0.474	0.073	0.084	0.565	0.796	0.806	0.470
**Vit B_3**	[mg]	*r*	-0.029	**-0.549**	-0.120	-0.047	0.131	0.405	0.104	-0.365	0.104	0.102
		p	0.905	**0.012**	0.613	0.845	0.582	0.077	0.663	0.113	0.663	0.688
**Vit B_6**	[mg]	*r*	0.101	-0.382	-0.027	0.117	0.304	0.343	0.379	-0.441	-0.248	-0.063
		p	0.673	0.097	0.910	0.622	0.193	0.139	0.099	0.052	0.292	0.791
**Vit B_{12}**	[μg]	*r*	-0.029	0.005	0.248	0.223	0.438	0.122	0.095	0.209	0.111	-0.008
		p	0.905	0.985	0.054	0.346	0.054	0.609	0.691	0.376	0.640	0.975
**Folic acid**	[μg]	*r*	0.229	-0.165	0.071	0.209	0.398	0.152	0.308	-0.257	0.101	-0.117
		P	0.332	0.486	0.767	0.376	0.082	0.523	0.186	0.274	0.673	0.622
